# Bedazzled: A Blue and Black Ship, Dressed to Deceive

**DOI:** 10.1068/i0727sas

**Published:** 2015-04-01

**Authors:** Tim S. Meese

**Affiliations:** School of Life and Health Sciences, Aston University, Birmingham, UK

**Keywords:** abstract expressionist art, blue and black dress, color constancy, cubism, dazzle camouflage, discounting the illuminant, misperception of color, painting, razzle dazzle, sundown, sunset, World War I, WWI

## Abstract

The blue and black dress that “melted the Internet” is thought to have done so because its perceived color depended on people using different prior assumptions about discounting the illuminant. However, this is not the first monochromatic object to have confused the public. For a brief period during WWI, RMS Mauretania was dressed in (dazzle) camouflage shades of blue and black/grey, yet she is sometimes depicted by artists, modelers, and historians in a much showier dress of red, blue, yellow, green, and black. I raise the possibility that this originates from a case of public deception deriving from the momentary misperception of a playful artist who neglected to discount the illuminant, propagating the most (perhaps only) successful application of dazzle camouflage known.

On 26th February 2015, a picture of a dark blue and black dress went viral on social media. The reason? When forced between two alternatives, about half of the population saw it as blue and black, the other half as white and gold (Figure 1). Broadly speaking, the generally accepted explanation is as follows: the low-grade photograph was overexposed and its chromatic content became seriously distorted, resulting in a spectrum that can be parsed in at least two different ways: (1) yellowish light from the front that when discounted leaves a dress made from blue and black material; (2) a garment in dim shadowy blue light that when discounted leaves a dress made from white and gold material. A third commonly reported visual interpretation is of a pale blue and brown dress where, presumably, the illuminant is not discounted at all (or is assumed to be made of flat white light), and the pixel wavelengths are seen to be (close to) how they look in isolation.

The details of all this, including the properties of the fabric and why different people have different priors for the image/illuminant, are being investigated in several vision laboratories across the world, and *Journal of Vision* is planning a special issue on the subject.

The purpose of this note is to bring to the attention of the scientific vision community a much older (~100 years) misperception of a rather different carrier of a blue and black (or gray) dress, possibly involving processes of the third kind described above. During the First World War, merchant ships and their escorts were painted in so-called dazzle camouflage consisting of crazy patterns of stripes and geometric shapes. Many schemes used black and white, but blues and pastel colors came to be used in some. It seems that the aim of this striking display was to dazzle the U-boat captains, confusing their assessment of the speed and heading of their targets (e.g., Scott-Samuel, Baddeley, Palmer, & Cuthill, 2011; Hall, Cuthill, Baddeley, Shohet, & Scott-Samuel, 2013). Given the large number of vessels involved (over 4,000 merchant ships and 400 military ships from Britain alone, according to Murphy & Bellamy, 2009), the application of dazzle must have impressed the authorities, but whether this strategy was truly effective in practice was never shown; it might well have been done more for morale than for saving allied shipping.

During WW1, the four-funnel liner RMS Mauretania was commandeered as a troop ship, and by 1918 she was dressed in dazzle camouflage. Photographs from this period are in black and white, and this has posed difficulties in assessing the true colors in which dazzle ships were painted, in general. The c1919 painting by Burnett Poole (Figure 2) shows Mauretania in a scheme of blues and grays. Whether these are the true shades that she carried, or whether the darker blue (such as that to the aft) was closer to black, as shown in Admiralty diagrams (held by the Imperial War Museum), is not clear. However, the camouflage shades of gray in every one of the photographs of the ship I have found on the Internet are consistent with each of these two (rather similar) interpretations. More importantly, I have found no evidence (photographic, eyewitness reports, or reference to Admiralty records) that Mauritania ever carried different coloration in any of the pale diamonds on the fore of the hull. Builders of scale models have a tenacious interest in the subject of livery/camouflage, and Internet posts from them come to similar conclusions. Nonetheless, an alternative scheme is readily found in reference books (Williams, 1989, 2001), paintings, (including one by Crossley) and Internet commentaries; it has exactly the same pattern as the established one, but includes large slabs of bright red on the hull and superstructure, and pastel shades of yellow and green on two of the funnels. So what is going on?

**Figure 1.**
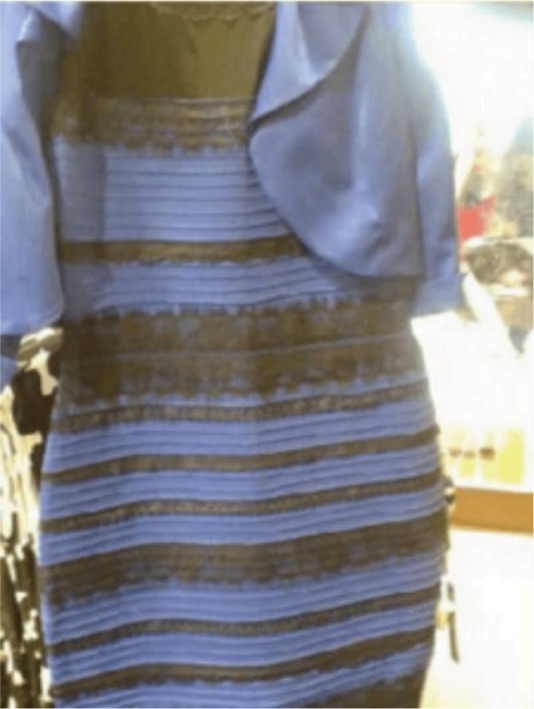
The blue and black dress that introduced the public to the fact that perception of color is not just about “reading” the RGB of each pixel. (This image is assumed to be public domain.)

**Figure 2.**
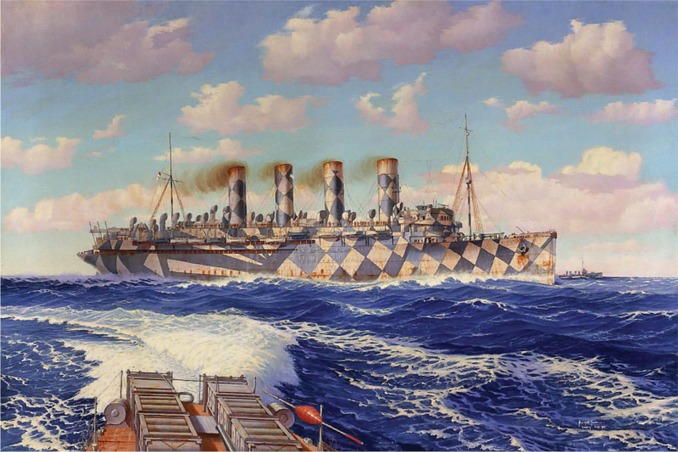
The blue and black ship? Admiralty diagrams (not shown) indicate that the official scheme was blue, black, and gray. (This image is public domain.)

**Figure 3.**
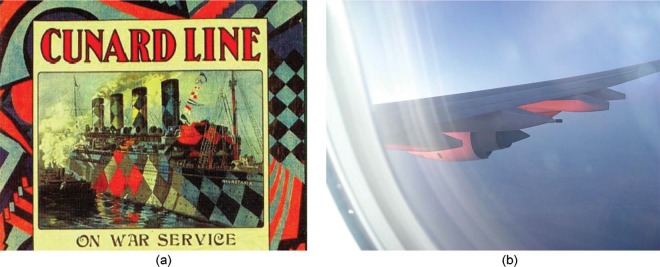
Seeing red. (a) Part of a post-war publicity poster for the Cunard Line. Almost certainly, this image is wrong in depicting RMS Mauritania in red, green, and yellow. This author does not know if the poster's true colors have been accidently/subtly distorted during reproduction, but even so, that could not explain the large splashes of red. Furthermore, the patchwork of gray in the original black and white photograph upon which this image was presumably based (not shown) is inconsistent with a grayscale conversion of this image (not shown). The speculation that the artificial colors were inspired by reflections at sunset is supported by the somewhat iridescent quality of some of the red regions in the image, particularly around the lifeboats. (This image is assumed to be public domain.) (b) Photograph of the wing of an Airbus taken at sundown. A dazzling spectacle, since commercial airlines do not usually paint the underwings of their aircraft red or pink! (In fact, knowledge of this might impede perception of a colored underwing (e.g., Hurlbert & Ling, 2005; Granzier & Gegenfurtner, 2012). (This image is public domain.)

The origin of this (assumed) error is almost certainly a post-war publicity poster by the ship's owners, The Cunard Line, which depicts their liner (Figure 3a) in the exact alternative colors described above; it seems that these have been taken on face value by those who have seen the poster, and the myth has been propagated. But the question arises, why gild the lily? The answer might be that the more colorful scheme was simply thought to be more appealing to potential passengers; but why choose *those* colors? After all, there is little evidence that bright red was used in British dazzle schemes; available records suggesting that more sombre maritime colors were by far the norm. (The Imperial War Museum website hosts images of (at least) 2,185 scale models, photographs, paintings, and drawings of dazzle ships, many carrying the curiously named Wilkinson schemes (e.g., 9A, 9EX, 11EX, 14G, 19, and 24-11); in the rare cases where shades of red can be found, they tend toward bauxite or red oxide, not scarlet). We can't know for sure, but it is not so fanciful to suppose that the artist of the publicity poster had seen Mauritania (or other dazzle ships) in port at sunset; reflections of red, and arguably yellow and green, being directed to the artist by some of the paler (more reflective) facets of the ship (a contemporary example is shown in Figure 3b). With a cognoscente, or even naïve, prior that *anything is possible with these dazzle ships*, these splashes of narrowband illuminant might well have been perceived as paint, at least for a few moments, and—enjoying the spectacle—the artist chose to share that with the public, in a stylized fashion.

Whatever the underlying reason for this misperception of the blue and black ship, it has proved provocative (e.g., the contemporary artwork in Figure 4^1^), and it is delightfully ironic that what is probably dazzle's greatest success in deception has nothing to do with the purpose for which it was intended; perhaps also, that a similarly misperceived blue and black dress should be the trigger for Mauritania's public disrobing.

**Figure 4.**
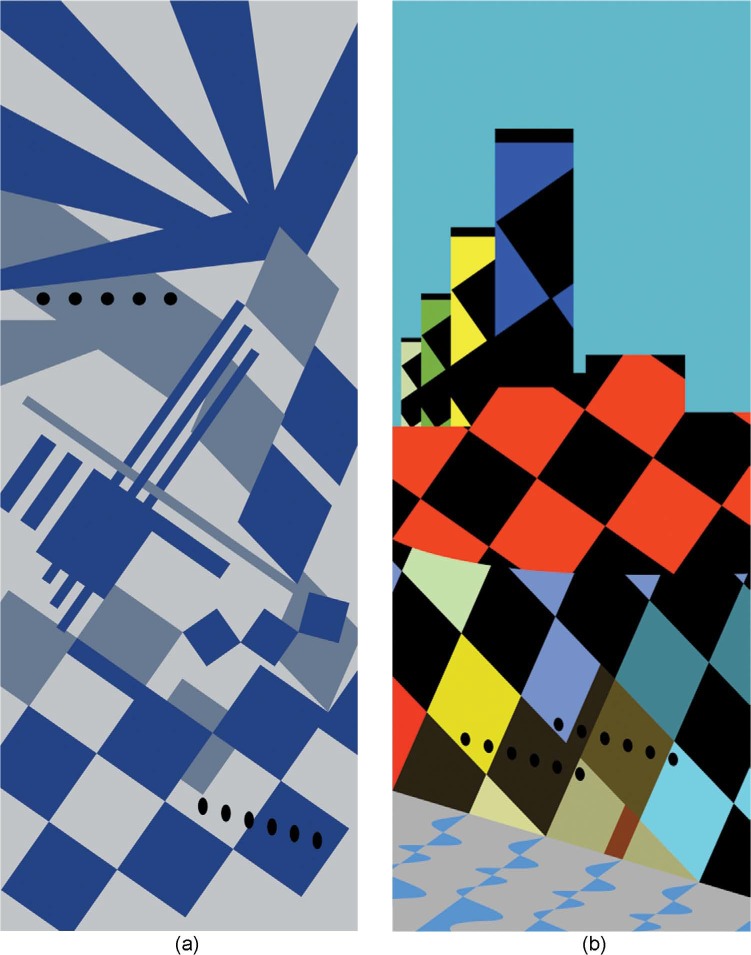
The door to misperception: Abstract expressionist artwork (2011) inspired by the dazzle camouflage of RMS Mauritania, printed on each side of a domestic internal door. (a) An image inspired by (i) the different dazzle patterns found on the two sides of the ship, (ii) the painting by Burnett Poole (Figure 2). and (iii) a model constructed by Jim Baumann (not shown). (b) The “sunset” colors were inspired by those found in the Cunard Line publicity poster (Figure 3a) in an image that also pays homage to the cubist art period of the time (note the orthogonal directions of travel implicit in the superstructure). (Copyright held by the author/artist.)

## Footnotes

1 It is often thought that the cubist art of the period was the inspiration for the dazzle schemes of WW1. However, the original proposals by Kerr were derived from observations of animal camouflage with the help of Thayer (Murphy & Bellamy, 2009), and it has been claimed that Wilkinson's schemes were derived quite independently of the fashionable art movement of the time, though experiments with cubist ideas were carried out by the French *sections de camouflage* (Murphy & Bellamy, 2009).
